# A Systematic Approach to Discover and Characterize Natural Plant Biostimulants

**DOI:** 10.3389/fpls.2016.00435

**Published:** 2016-04-05

**Authors:** Giovanni Povero, Juan F. Mejia, Donata Di Tommaso, Alberto Piaggesi, Prem Warrior

**Affiliations:** Global R&D Department, Valagro SpAAtessa, Italy

**Keywords:** abiotic stress, *Ascophyllum nodosum*, biostimulants, crop growth and development, plant nutrition, seaweeds

## Abstract

The use of natural plant biostimulants is proposed as an innovative solution to address the challenges to sustainable agriculture, to ensure optimal nutrient uptake, crop yield, quality, and tolerance to abiotic stress. However, the process of selection and characterization of plant biostimulant matrices is complex and involves a series of rigorous evaluations customized to the needs of the plant. Here, we propose a highly differentiated plant biostimulant development and production platform, which involves a combination of technology, processes, and know-how. Chemistry, biology and omic concepts are combined/integrated to investigate and understand the specific mode(s) of action of bioactive ingredients. The proposed approach allows to predict and characterize the function of natural compounds as biostimulants. By managing and analyzing massive amounts of complex data, it is therefore possible to discover, evaluate and validate new product candidates, thus expanding the uses of existing products to meet the emerging needs of agriculture.

## Introduction

One of the biggest challenges for agriculture is the development of sustainable and environmentally friendly systems to address the need to feed the growing world population. With decreasing area of arable land as we approach the limits of genetic potential of staple crops, the only way to achieve this objective is by increasing the crop yield and protecting what we produce. In other words, produce “more with less" (Food and Agriculture Organization of the United Nations [FAO], 2012; Carvalho and Vasconcelos, 2013; International Food Policy Research Institute [IFPRI], 2014). Parallel to this, reducing energy consumption and utilizing resources more efficiently should be priorities (Gregory and George, 2011). Simultaneously, quality of crops should be enhanced, particularly under unfavorable growing environments. This means obtaining higher incomes for farmers, having better postharvest storage and more nutritious food for consumers (Eckardt et al., 2009).

One of the most innovative and promising solutions to address these important challenges consists of the use of plant biostimulants (PBS), referred as “*materials which contain substance(s) and/or microorganisms, whose function when applied to plants or the rhizosphere is to stimulate natural processes to enhance/benefit nutrient uptake, nutrient efficiency, tolerance to abiotic stress, and/or crop quality, independent of its nutrient content*” (European Biostimulant Industry Council [EBIC], 2016).

**FIGURE 1:**
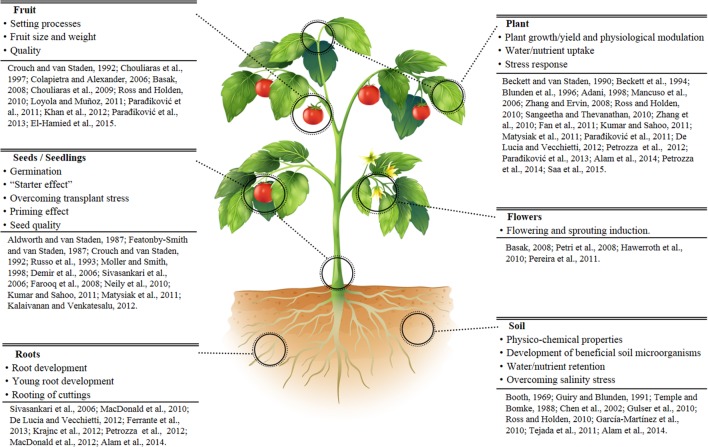
**Reported examples of the main effects and physiological actions played by plant biostimulants (PBS)**.

Plant biostimulants formulations are generally proprietary compositions based on seaweed extracts, complex organic materials, plant hormone-like compounds, amino-acids, and humic acids. Extensive reviews discuss the large group of PBS derived from seaweeds, in particular *Ascophyllum nodosum* (Khan et al., 2009; Craigie, 2011; Calvo et al., 2014; Sharma et al., 2014) and the beneficial effect of natural biostimulants on specific aspects of plant growth, production and fruit quality in different crops (Paradjiković et al., 2011, 2013; Bulgari et al., 2015; Saa et al., 2015). Specific PBS activities such as increased root and shoot growth, tolerance to abiotic stress, water uptake, reduction of transplant shock etc., have been also reported (Adani et al., 1998; Parrado et al., 2008; Alam et al., 2014; Petrozza et al., 2014). Biostimulants can also reduce fertilizer use and nutrient solution concentration in hydroponic systems (Vernieri et al., 2006). A summary of the beneficial effects of PBS is reported in **Figure 1**.

Considering the above, we expect a critical role for biostimulants in the agriculture of the future. The market of biostimulants is estimated to worth $1,402.15 million in 2014, and is projected to reach $2,524.02 million by 2019, at a Compound Annual Growth Rate (CAGR) of 12.5%. The expected drivers of this growth include (i) growing importance for organic products in the agriculture industry (ii) increase of biostimulants application in developing countries (iii) more global PBS presence and acceptance among customers, since the players in this market have developed a range of innovative products to satisfy specific crop needs (Biostimulant Market, 2014).

While the knowledge on the benefits of PBS on plants is steadily improving – evidenced by a significant increase of research papers focused on PBS – there has been little attention to the critical scientific steps required for an optimal selection and characterization of biostimulant compounds, based on chemical and biological analyses to develop optimal solutions for specific agronomical needs.

Here we propose a robust platform that we named GeaPower^®^, based on different research approaches and a combination of technology, know-how and processes consolidated over a decade of experience aimed to investigate and develop effective PBS products.

## From Raw Materials to Biostimulants: A Step-By-Step Procedure

### When the Process Begins: Definition and Analysis of an Unmet Need

The first step that triggers the research platform for the study of new PBS formulations is the definition of a specific agricultural need. The study of the desired attribute along with a thorough review of scientific literature in consultation with scientific experts in the research field of interest, allows us to draw a list of the natural sources or active ingredients that may be included in a future prototype. An in-depth understanding of the biological and chemical characteristics of raw materials such as seaweeds, microorganisms and their metabolites, plant extracts, is needed to identify, characterize and preserve specific active ingredients that can help achieve the targeted physiological responses in plants. Thus, it becomes crucial to choose the right time and season to obtain -from raw materials- the most optimal yield of specific biomolecules needed for researched activities (Parys et al., 2009; Apostolidis et al., 2011). A typical example of this approach is the use of *Ascophyllum nodosum* (L.), one of the most researched seaweeds (Ugarte et al., 2006), and one of the raw materials utilized in the GeaPower^®^ technology. It is recognized as the dominant intertidal seaweed of the North Atlantic coastline where water temperatures do not exceed 27°C (Keser et al., 2005), although this alga is known to grow under extreme temperatures, from -20°C in winter to air temperatures of 20–25°C and direct sun heating in summer (Strömgren, 1983).

*Ascophyllum* can be collected in Norway, where this macroalga is exposed to 6 months of darkness during polar night, but also subjected to high solar radiation in spring, especially during low tide and high water transparency, leading to strong oxidative stress due to the formation of reactive oxygen species (ROS) induced by environmental factors (Dummermuth, 2003; Di Tommaso, 2012). These extreme conditions confer pliability, elasticity, ability to conform to the flow, and influence the chemical composition of this seaweed, as a consequence of the exposure degree (Black, 1948, 1950; Biber, 2002; La Barre et al., 2004). Careful selection and harvest of algal materials with select biological attributes thus becomes extremely critical to developing select biostimulant preparations.

### Extraction, Chemical/Biological Characterization, and Prototyping

Customized extraction processes are required to maintain a precise ratio of each ingredient in complex natural mixtures thus assuring the efficacy, quality, and consistency of the final products.

Extraction procedures are calibrated in order to selectively isolate categories of chemicals specific for the intended use, utilizing appropriate solvent mixtures, pH, temperature and eventually enzymes to drive the process. The challenges and hurdles in these procedures have been very well described by Harborne (1984). Hou et al. (1997, 2000) described methods to isolate alginate-containing fractions, pigments, proteins, and sulphated polysaccharides, including specific processes for the highest possible yield. Enzyme digestion procedures can also be used in several processes to shorten the polymer length, for example in proteins, but also in polysaccharides, resulting in enhanced biological activity and bioavailability (Jiménez-Escrig et al., 2011).

Further, the natural extracts must be analyzed qualitatively and quantitatively for actives that they may contain. According to the specific molecule (or family of molecules) that needs to be analyzed, one may choose liquid chromatography, such as HPLC-DAD-FLD, LC-MS-MS, Q-ToF, or gas chromatography, with GC-MS. The first step is the identification of the compounds of interest, using qualitative techniques, such as GC-MS, LC-MS-MS, Q-ToF. After this challenging step, the development and validation of analytical methods for each active ingredient to be quantified enables the appropriate separation of the biomolecules of interest from other molecules present in the background, thus allowing the realization of calibration curves and quantification, ensuring minimal batch-to-batch variation.

If, for example, we consider an alkaline extraction of seaweeds, alginates will be partially converted to carboxylic acids (Niemela and Sjostrom, 1985) and their identification and quantification can be carried out by GC-MS; during the same extraction procedure, polyphenols, such as phloroethols, will be rearranged as complex dibenzofurans (Ragan and Glombitza, 1986), and HPLC-DAD will be a good analytical tool to separate, qualitatively identify and quantify them against a suitable internal standard. Fucoidans will undergo hydrolysis in oligomers and constituent sugars. After complete hydrolysis and derivatization, each monosaccharide can be quantified with HPLC-DAD. Betaines presence can be quantified with LC-MS-MS (Blunden et al., 2010), and the structure of native phlorotannins can be elucidated by NMR (Parys et al., 2007), but also by Q-ToF (Tierney et al., 2013).

Once the desired combination of active ingredients is defined, it is very important to check and match the regulatory guidelines on the different crops and geographies where they are intended to be used. Simultaneously, a primary evaluation of the formulation for the safety profile should be performed. The technology to realize prototypes in liquid, emulsion, microgranular and powder form is required to address the market needs; often these could be the greatest challenge in biological formulations and is generally considered proprietary information at the manufacturer level. Standard shelf-life and stability trials including the accelerated aging conditions, in warm (45°C) and cold environments (+4, -4, and -20°C) on each prototype in the final packaging are carried out. Following these evaluations, a detailed profile of the active ingredients evolution over time and changes in physical parameters of each prototype under each aging condition is performed to develop recommendations for appropriate shelf-life and storage conditions.

### Biological Screening

Chemical analyses are further substantiated by biological assays that characterize the composition at different levels, including the physiological mechanisms activated by specific compounds. This is essential to enrich the internal library, a proprietary database including all the information about molecules of interest, to link each component to a specific function and use this information to better set prototype formulations according to the need.

It has become increasingly evident that understanding the functional links between genes/transcripts, proteins, metabolites and nutrients is one of biology’s greatest challenges, and recent technological improvements have brought major advances in this area (Carvalho and Vasconcelos, 2013). Functional genomics presents a powerful tool that allows us to decipher the molecular and physiological triggers for specific responses in plant systems (Bouchez and Höfte, 1998). DNA microarrays are clear examples of functional genomic application: a high-throughput technology that allows rapid and quantitative measurement of parallel expression of thousands of genes (Aharoni and Vorst, 2002). The transcriptomic profiling provided by microarrays datasets can generate a picture of cellular functions under a given experimental condition (Schena et al., 1995; Tan et al., 2009). In the field of PBS also, the use of microarrays has allowed us to dissect the effects of PBS at transcriptomic level, highlighting the ability of raw materials that are used to formulate PBS to induce the expression of various sets of genes. By using molecular tools, it is possible to hypothesize possible modes of action of different substances, predicting their role as biostimulants (Santaniello et al., 2013).

**FIGURE 2:**
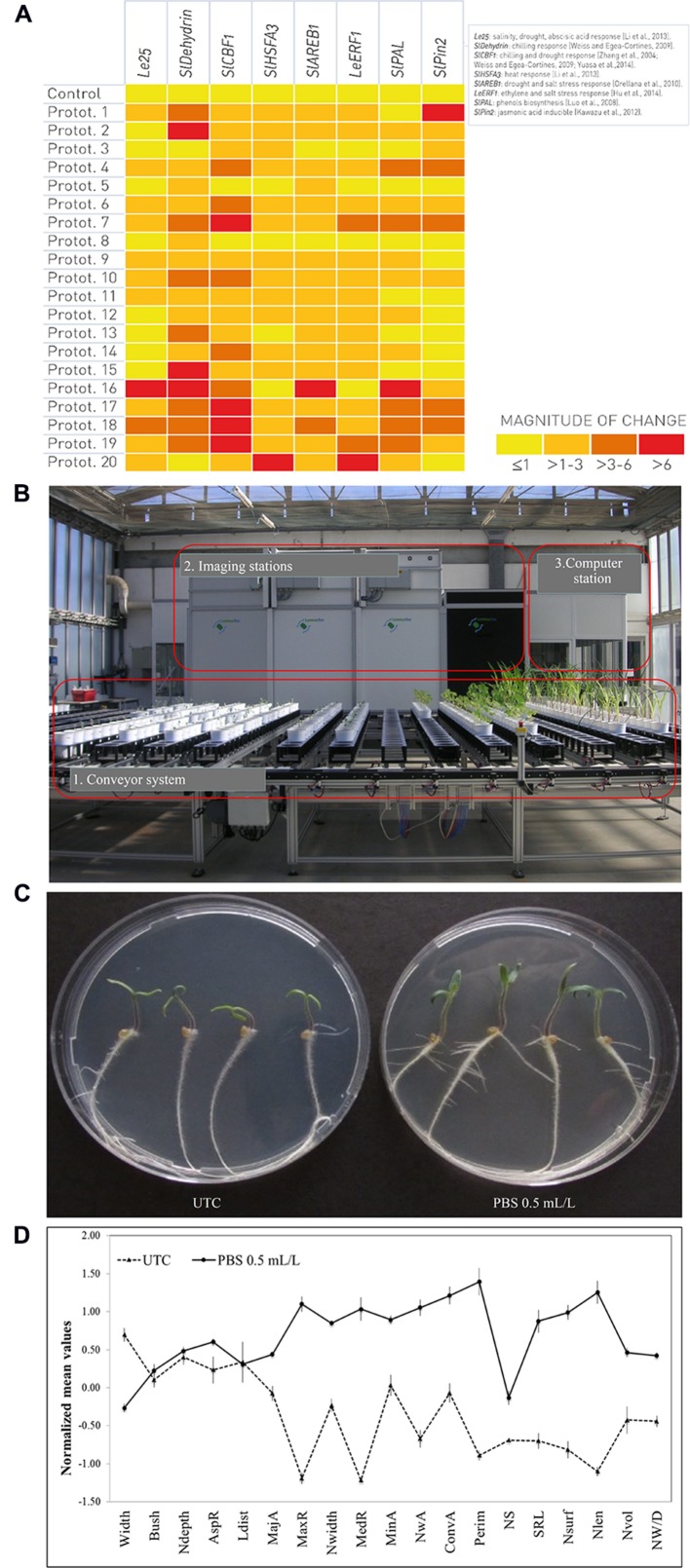
**Genomic, phenomic, and *in vitro* platforms to screen the effect of a set of different natural extracts-based prototypes using tomato (*Solanum lycopersicum* L.) cv. Microtom as plant model. (A)** Relative abundance of mRNA transcripts of markers for specific physiological processes in response to a range of treatments with biostimulant prototypes (Protot.). Transcript levels (signal intensities) are presented in the form of a heat-map (using HeatMapper Plus Tool), on a color scale between low (yellow) and high (red). **(B)** LemnaTec-Scanalyzer 3D System placed at ALSIA – Metapontum Agrobios Research Center (Matera, Italy). The physiological and morphometric parameters that can be measured using plant phenomics are UV (Ultraviolet) to fluorescence, photosynthesis and health index; RGB (Red-Green-Blue) to plant morphology, architecture, digital biomass and green/yellow index; NIR (Near-Infrared) to plant water content. **(C)** Comparison of UTC (untreated control) and PBS-treated (final concentration of 0.5 mL/L) plants grown on agar-based medium. **(D)** Root phenotype differentiation, based on imaging analysis software GiA Roots (Galkovskyi et al., 2012), comparing UTC (untreated control) with PBS-treated (final concentration of 0.5 mL/L) plants. Traits displayed are: Average root width (Width), Bushiness (Bush), Network Depth (Ndepth), Aspect ratio (AspR), Network length distribution (Ldist), Major Ellipse Axis (MajA), Maximum number of roots (MaxR), Network width (Nwidth), Median number of roots (MedR), Minor Ellipse Axis (MinA), Network Area (NwA), Network Convex Area (ConvA), Network perimeter (Perim), Network solidity (NS), Specific root length (SRL), Nsurf (Network surface area), Network length (Nlen), Netwok volume (Nvol) and Network width to depth ratio (NW/D). The error bars indicate the standard error of the mean.

Besides the so called transcriptomic fingerprint released by a microarray analysis, a parallel and/or subsequent qPCR study is often needed in order to validate the data, starting from a microarray dataset or *de novo* selection of target genes involved in specific physiological processes (Morey et al., 2006). For example, to screen the effect of a set of different seaweed-based prototypes, we selected a set of tomato genes involved in different biochemical pathways that can be used as markers for the qPCR screening (**Figure 2A**). If one or more gene markers display a significant differential expression after the application of a prototype in comparison with untreated control, then it is possible to hypothesize a role of the prototype in the physiological process(es) in which the gene(s) is/are involved. For instance, this can suggest a possible priming effect of treated crops against distinct stresses (**Figure 2A**). More generally, the list of genes can be chosen and/or extended according to the needs and/or desired results, and also the experimental conditions (e.g., normal vs. stressful conditions) can be chosen based on the target.

In parallel with genomics, the phenomic approach permits the study of PBS on plant growth, performance, and composition based on multi-spectrum, high-throughput image analysis to detect morphometric and physiological parameters (Furbank and Tester, 2011). Such multi-spectrum analysis (infra-red, visible, and ultraviolet light) of reflected or re-emitted light from the plant crown, stem and leaves provides information on the nutritional, hydrological and physio-pathological state of plants, as well as on a plant’s ability to absorb light (Petrozza et al., 2014, **Figure 2B**). One example of a phenomic facility is the high-throughput plant phenotyping platform (LemnaTec-Scanalyzer 3D system) placed at the ALSIA Centro Ricerche Metapontum Agrobios s.r.l. (Matera, Italy; **Figure 2B**; Petrozza et al., 2013).

The value of an integrated molecular/phenomic analysis in characterizing the role of a specific PBS was recently demonstrated in relation to the application of Megafol^®^, a natural PBS, on tomato plants in order to show the reduction of drought-stress related damage (Petrozza et al., 2012, 2014).

Besides omics, *in vitro* assays may also be useful tools to speed up the process of preliminary screening. Here, plants are germinated under sterile conditions in petri dishes, flasks, or tubes, and then grown on a liquid or a solid medium in an incubator, where light and temperature parameters can be modulated and monitored. According to the plant model, PBS formulations are added either to the solid or the liquid medium at different concentrations to evaluate dose-effect response curve. Such experimental conditions allow us a fast screening of prototypes on plants, eliminating the influence of soil and other environmental parameters, including competition with fungi and bacteria (Chawla, 2004). For example, it is possible to perform *in vitro* tests using tomato (cv. Microtom) seeds on an agar-based substrate containing sucrose 1.5%, without any additional nutrient sources. A PBS formulation may be included into the medium, in order to evaluate the biostimulant effect of selected matrices, in relation to untreated controls (**Figures 2C,D**).

### Tests in Controlled Environment

It has been demonstrated that the use of PBS can improve quantitative and qualitative parameters also if applied in hydroponic or other environmentally controlled crop systems. Some of the effects reported are the reduction of nutrient solution concentration in floating system, besides yield and nutritional quality increase. For this reason, screening under plant growth chambers and greenhouses are considered robust methods to evaluate the agronomical validity of PBS formulations (Vernieri et al., 2006; Paradjiković et al., 2011). Plants are grown directly on soil, pots, or liquid media (hydroponic solution), and treated with prototypes (foliar and/or root applications). This approach allows us to define the best application methods, timing and rates, and provides preliminary indications on phytotoxicity. The use of the plant growth chamber is ideal for studies of specific kinds of stress, such as temperature stress, giving quantitative and qualitative evidences related to the compounds tested (Feng et al., 2003).

In conclusion, the above described steps, provide robust scientific bases to support the development of innovative PBS solutions for agriculture.

### Product Development: Phytotoxicity Assessment, Field Testing, REACh Compliance

Even if certain prototypes display good efficacy in respect to the initial need, other crucial factors need to be considered before releasing a new commercial product, in particular:

#### Phytotoxicity

In order to assess any negative/toxic effects of the selected prototype on plants, several phytotoxicity tests are carried out on different target crops, using a large range of rates of application.

#### Field Trials

Once the agronomic performance of a certain prototype is determined, it is critical to validate them through a number of trials under field conditions. In order to ensure robust and statistically significant results, the efficacy of prototypes should be accurately verified on target crops worldwide. Thus, prototypes are tested in varying agroclimatic conditions, under distinct growing environments and according to local agronomic practices.

REACh (Registration, Evaluation, Authorisation and Restriction of Chemical substances for the European Union) compliance is critical: in accordance with the EU guidelines for agricultural product development, the preliminary safety evaluation is integrated with the REACh compliance assessment that includes physicochemical, toxicological and ecotoxicological evaluations. Under REACh it is necessary for producers/importers to register chemical substances unless exempt from REACh registration (European Parliament, Council of the European Union, 2006). In case of the use of microorganisms, a particular dossier should be filled out, in order to specify the identity, properties (or characteristics), toxicology and other attributes of the selected microbials for the intended use as PBS (Kamilova et al., 2015).

### The Last Step: Process Development and Further Scientific Validation

Once a new PBS prototype is selected according to the Geapower^®^ steps described earlier, the commercial team embarks on developing a manufacturing process which is efficient, consistent and optimizes yields and costs. This step is carried out at the lab and pilot plant facilities (upstream and downstream equipments), to simulate commercial scale up. At this point the prototype may be launched as a pre-commercial prototype, which becomes available for the scientific community to perform further scientific studies. This is for example the case of a biostimulant developed to overcome abiotic stress such as drought, composed of specific amino acids, glycosides, vitamins, polysaccharides, betaines, organic nitrogen and carbon derived from *Ascophyllum nodosum* and other plant materials (Saa et al., 2015). Plants pre-treated with this PBS were healthier in terms of digital biomass (image-based biomass estimation) and chlorophyll fluorescence, and this positive effect was confirmed at molecular level observing a lower expression of drought-related genes even when plants were strongly water-stressed. This suggests that treated plants were indeed experiencing a lower level of water stress, as a consequence of the treatment itself (Petrozza et al., 2014). In addition to this case study, the beneficial attributes of PBS in recent years has resulted in an increasing number of research papers that validate the commercial value of these complex natural compounds. In our view, this will be bring important advances in the detection and demonstration of clear, measurable effects of PBS on plant production and, more generally, in agriculture.

## Conclusion

The increasing pressure on the land to support a fast-growing world population has made it necessary to intensify agricultural production. Such pressure will inevitably lead to the development of alternative technologies to improve the efficiency of crop production and food security (Beddington, 2011). The solution we propose involves a highly differentiated PBS discovery, development, characterization, and production platform, to which we give the name GeaPower^®^. This approach uses the power of chemistry, biology and omics to integrate large amounts of complex data in order to assess and validate the inherent activities and synergies of candidate natural compound mixtures and micro-organisms for commercial use in agriculture.

We believe this systematic approach, starting from customized access to raw materials, through extraction methods to product development, helps efficiently turn prospective natural active ingredients into high quality nutrient solutions. GeaPower^®^ permits us to understand what makes a PBS formulation work explaining the mode(s) of action of complex biomolecules, and discover new opportunities. With this approach it is also possible to predict the function of natural substances and how they modulate the physiology of plants, making them more efficient even under limited water and/or nutrient resources in their environment (du Jardin, 2012).

Finally, in our view extensive experience with field trials, together with continuous research and know-how acquisition in terms of PBS formulation and biological effect, are and will be crucial to satisfy the needs of present and future professional agriculture. The plant biostimulants may thus represent the previously non-existent bridge between the biological, live products and the prescriptive chemical products that serve the agricultural input markets.

## Author Contributions

GP: ideation of the manuscript; main writing and structuring of the paper; physiology/genomics topics inside the manuscript. JM: introduction section; topics related to microorganisms; figures. DT: contribution in the Introduction section, especially on algae as biostimulants; chemical characterization and product development paragraphs; AP: biological characterization section; critical review. PW: conclusion and critical review of the entire manuscript; figures choice.

## Conflict of Interest Statement

The authors declare that the research was conducted in the absence of any commercial or financial relationships that could be construed as a potential conflict of interest.
